# Size- and density-dependent gastric emptying of emulsion-alginate beads for tailored *in vitro* intestinal lipolysis

**DOI:** 10.1016/j.crfs.2025.101279

**Published:** 2025-12-15

**Authors:** Lingfeng Wu, Karin Schroën, Meinou Corstens

**Affiliations:** Wageningen University, Department of Agrotechnology & Food Sciences, Laboratory of Food Process Engineering, Bornse Weilanden 9, Wageningen, 6708 WG, the Netherlands

**Keywords:** Controlled release, Encapsulation, Particle size, Gastric emptying, Intestinal lipolysis, *In silico* modelling

## Abstract

The gastric emptying profile is essential for nutrient digestion and metabolism, and its complex relation with food structure is not well understood. Advanced dynamic *in vitro* digestors in combination with model food structures offer promising ways to study and control digestion in the gastrointestinal tract. In this study, the NEar Real Digestive Tract (NERDT) system is used to investigate the impact of gastric emptying on intestinal lipolysis of emulsion-alginate beads. These model food structures were 0.8–3.2 mm in size, and had different density.

All particles were strong enough to resist the applied peristaltic forces and remained intact throughout gastrointestinal digestion. Particles made with higher alginate concentration settled down in the stomach due to higher density, leading to a prolonged lag time in gastric emptying. Particles with a density similar to gastric fluid mix more homogeneously and are emptied faster, depending on their as determined by gastric sieving. The half gastric emptying time (*t*_*0.5*_) varied between 43.8 and 112 min, with higher gastric homogeneity corresponding to faster emptying, that is when particles were small and not prone to gastric sieving. The gastric emptying profiles were used to predict the intestinal substrate concentrations and FFA release profile in time. The experimental data and model showed that ∼70 % of the fatty acid release takes place in the duodenum. These insights support food matrix design for controlled digestion in the small intestine.

## Highlights

• Larger density and size delayed the gastric emptying of emulsion-alginate beads.
• Gastric emptying determines the intestinal FFA release profile.
• Static lipolysis kinetic can be used to predict dynamic intestinal lipolysis.
• The Near Real Digestive Tract (NERDT) is used for dynamic intestinal lipolysis.

## Graphical abstract

**Figure:**
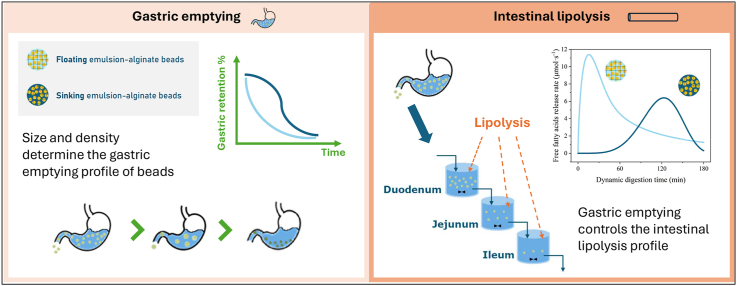

## 1 Introduction

Oils are a source of vitamins and essential fatty acids that may create health effects, such as anti-inflammation (Salsinha et al., 2021), but overconsumption of oil increases the risk of metabolic disorders such as obesity, hyperglycaemia, and hyperlipidemia (WHO, 2023) due to its high energy value. Besides, oil can also encapsulate lipophilic bio-active compounds (Ahmed et al., 2012; Kaimainen et al., 2015). During gastrointestinal (GI) digestion, the food structure in which the oil is present plays a complex role. It influences how fast a food is passed through the GI tract, and through that the digestion profile that in turn is determined by both biochemical conditions (enzymes, acids, electrolytes) as well as physical forces (peristalsis, compression) (Dupont et al., 2019). Consequently, a comprehensive investigation into the mechanism of oil digestion must address both enzymatic kinetics and the transit of oil throughout the GI tract.

Among the various physiological factors affecting lipid digestion, gastric emptying has emerged as a key modulator (Goyal et al., 2019). Food emptied from the stomach has undergone several phases: tonic and peristaltic contractions, retropulsion, mixing, grinding and removal from the stomach (Goyal et al., 2019; Liu et al., 2021). After the contractions, food is pushed to the distal stomach and mixed with gastric fluid by pylorus contraction, after which the pylorus passes small enough food particles (sieving) to the duodenum: the actual emptying process (Liu et al., 2021). Gastric sieving can separate a homogeneous soup into solid and liquid parts and retain the solid food longer in the stomach (Marciani et al., 2012).

*In vivo* studies have shown that the characteristics of foods or drugs affect gastric emptying behaviour, and this may also affect the oil digestion profile (Kong and Singh, 2008b). In general, most of the oil digestion happens in the small intestine (up to ∼80 %) (Armand, 2007). After oral processing, solid food is broken down into particles. Therefore, it is important to link the gastric emptying behaviour of food to its size and density, as these largely influence its gastric emptying profile (Bürmen et al., 2015; Clarke et al., 1995; Meyer et al., 1985, 1988; Podczeck et al., 2007; Tuleu et al., 1999), potentially due to the sieving action of the antrum and pylorus. For, example, the lag time of gastric emptying can be significantly longer, as demonstrated for solid egg white particles (2.5–5 mm) compared to smaller homogenized particles (Urbain et al., 1989). Additionally, it has been reported that substrate content (Deng et al., 2023), hardness (Deng et al., 2023; Marciani et al., 2001); viscosity (Sanggaard et al., 2004), caloric density (Liu et al., 2021; Salden et al., 2015) and phase separation (Camps et al., 2017) influence the gastric emptying of both liquid and solid food. These studies clearly show that gastric emptying is influenced by the meal consumed alongside the target particles (viscosity and the ratio of food to liquid).

Studying these effects in humans is inherently difficult, and from an ethical perspective often impossible (Dupont et al., 2019). To develop mechanistic insights, advanced standardized *in vitro* models are crucial for enhancing repeatability and comparison between studies, and correlation with *in vivo* data (Dupont et al., 2019; Minekus et al., 2014). Different dynamic digestors were developed to simulate the gastrointestinal tract (Dupont et al., 2019), such as dynamic gastric model (DGM) (Wickham et al., 2012), DIDGI® (Kong and Singh, 2008a) and TIM-1 (Nabil et al., 2011). These devices mimic the biochemical conditions of the GI tract (i.e., dynamic pH profile and enzyme secretion), and in some cases also the transit. For instance, the DGM creates antral shear rates and shear forces as *in vivo* calibrated with agar beads and controls the gastric emptying by antral sieving (Wickham et al., 2012). The NEar Real Digestive tract digestor (NERDT) comprises of a J-shaped silicone human stomach, motors, rollers, etc. to simulate peristaltic contractions, and emptying (Wang et al., 2019), thus generating realistic mechanical forces and gastric emptying (Elashoff's model) (Elashoff et al., 1982). The NERDT is able to accurately simulate the gastric emptying and digestion of cheese and protein (Feng et al., 2024; Peng et al., 2021), but the intestinal part has been explored less. Since it does play a crucial process in lipid digestion (Ko et al., 2020; McClements et al., 2009), we have made the connection of gastric emptying and intestinal digestion the focus of our paper. As mentioned earlier, food structure is expected to play a prominent role in gastric emptying, that is why we work with model structures to elucidate this connection.

Lipid digestibility and free fatty acid (FFA) release from emulsions (Tan et al., 2020) and emulsion-gels (Sabet et al., 2022) have mostly been studied using static *in vitro* models (Brodkorb et al., 2019; Minekus et al., 2014). Calcium-alginate hydrogels have been shown to protect oil droplets during gastric digestion (Corstens et al., 2017) allowing them to release during intestinal digestion, depending on their size, mesh size and integrity (Corstens et al., 2017; Li et al., 2011; Wu et al., 2024). What has not been taken into account yet, is how gastric emptying affects the release of lipolysis products in time.

In the current study, we use the NERDT to simulate dynamic gastric emptying to understand the effect of size and density of emulsion-alginate beads on their gastric emptying profile, and through that on the lipolysis profile. These profiles were compared with models that include both emptying profiles and enzyme kinetics to thus arrive at a deeper understanding of the role of the food matrix in lipid digestion.

## 2 Materials and methods

### 2.1 Materials

Sodium alginate (Protanal® GP8133) was kindly supplied by International Flavors & Fragrances (New York, USA). Safflower oil (that was shown to create the biggest satiety effect when used in *in vivo* experiments (Maljaars et al., 2009)) was purchased from De Wit Specialty oils (19,200 Safflower Oil High Linoleic Refined, The Netherlands). Whey protein isolate (WPI) was provided by Davisco Foods International (BiPro, Eden Prairie, Minnesota, USA; purity 97.5 %). The electrolyte composition of SGF and SSF, including KCl, KH_2_PO_4_, NaHO_3_, NaCl, MgCl_2_(H_2_O)_6_, (NH_4_)_2_CO_3,_ CaCl_2_(H_2_O)_2_, pepsin from porcine gastric mucosa (P7125), were purchased from Sigma Aldrich (St. Louis, MO, USA). Heptane (34873) was purchased from Sigma Aldrich (St. Louis, MO, USA). n-Hexane was supplied by Actu-All Chemicals (Oss, The Netherlands). 2-propanol was from Carlo Erba Reagents S.A.S. (Val de Reuil Cedex, France). tristearin (33–1800), 1,2-distearin (32–1802), 2-monostearin (31–1802) and 9(Z),12(Z)-octadecadienoic acid were purchased from Larodan (Retzius väg, Solna, Sweden) and used as standards. For all experiments, ultrapure water was used (Millipore Corporation, Billerica, Massachusetts, USA).

### 2.2 Methods

#### 2.2.1 Preparation of emulsion-alginate beads

The emulsion-alginate beads were prepared by dripping emulsion-containing alginate solutions into a calcium bath as previously reported (Wu et al., 2024). In short, 20 wt% O/W emulsions were prepared (*d*_*32*_ = 18.2 μm) by homogenizing a WPI solution (1 wt% dissolved in 10 mM phosphate buffer pH 7.0) and safflower oil (weight ratio 4:1) using a rotor stator homogenizer (IKA T18 basic Ultra-Turrax homogenizer equipped with a S18N-19G dispersion tool, Staufen, Germany) for 5 min at 10,000 rpm. Freshly prepared emulsions were mixed with alginate solutions (1.8, 3.6 and 5.4 wt% alginate solutions in 10 mM phosphate buffer pH 7.0, kept overnight under stirring) in weight ratios of 1:1 to have a final oil content of 10 wt% at 1, 2 and 3 % of alginate. After 30 min of mixing at 500 rpm, the emulsion-alginate mixture was pushed through a 0.41 mm inner diameter nozzle (Nordson EFD, UK) by a syringe pump (Harvard 2000, Holliston, Massachusetts, USA) at 400 mL/h with airflow at 1 bar to obtain small beads, and at 200 mL/h to obtain medium beads. Large beads are obtained by the similar process with a nozzle of 1.36 mm inner diameter (Nordson EFD, UK) without air flow. The formed droplets were captured in a 5 wt% CaCl_2_ bath to solidify them under gentle stirring. The beads were incubated in a 5 wt% CaCl_2_ bath for at least 60 min at room temperature before storing at 4 °C for 12 h. The beads were incubated in new 5 wt% CaCl_2_ at a ratio of 1:10 (g to mL) for 24h before use.

#### 2.2.2 Characterization of the emulsion-alginate beads

##### 2.2.2.1 Particle size

The diameter (*D*, m) of beads was determined for at least 50 beads by ImageJ 1.52. The diameters were calculated from their area (*A*) using Eq. (1).

Eq. 1 $$D=2\times \sqrt{\frac{A}{\pi }}$$

##### 2.2.2.2 Oil content

Firstly, emulsion-alginate beads were quickly washed in filter paper with Milli-Q® ultrapure water (MilliporeSigma, Burlington, Massachusetts, United States) at a 1:10 (v/v) ratio to remove free calcium. Then the surface water was removed by filter paper and 1g of beads were mixed with 15 g of n-hexane/2-propanol (3:2 (v/v) ratio) and disrupted by rotor stator homogenizer for 5 min at 15,000 rpm (IKA T18 basic Ultra-Turrax homogenizer equipped with a S18N-19G dispersion tool, Staufen, Germany). Then 5 mL ultrapure water was added to the mixture, and next centrifuged (10 min at 4000 rpm, 20 °C, ThermoScientific, Legend XFR) to speed up separation. Finally, the upper hexane phase was dried, and the measured oil amount, divided by the initial mass of beads, provided the oil content (Corstens et al., 2017).

##### 2.2.2.3 Density

The oil content of the beads was measured as described earlier. The density of oil (*ρ*_*oil*_, g·mL^−1^) and alginate (*ρ*_*ALG*_, g/mL) are 0.92 and 1.6 g mL^−1^, respectively. The density of water in the Ca-alginate gel is assumed equal to the CaCl_2_ solution (*ρ*_*w*_, ∼1.03 g mL^−1^) (Romanklw and Chou, 1983). Using the mass of oil and alginate (*M*_*alginate*_, g), the mass of water phase can be calculated from (Eq. (2)). Finally, the density of emulsion-alginate beads is determined from the volume fraction (*VF*) and densities of each component (oil, water, alginate) (Eq. (3)).

Eq. 2 $$M_{water\;phase}=M_{total}-M_{oil}-M_{ALG}$$

Eq. 3 $$Density\;of\;beads=\rho_{w}\cdot {VF}_{w}+\rho_{ALG}\cdot {VF}_{ALG}+\rho_{oil}\cdot {VF}_{oil}$$

##### 2.2.2.4 Strength

The fracture force of the beads was measured with a texture analyser (TA.XT Plus; Stable Micro System, Godalming, UK). Tests were performed with a cylinder probe with a diameter of 35 mm. The test speed was 1 mm/s and the trigger force was set at 0.1 g. Since the emulsion-alginate beads are quite soft, we used the fracture force as indication of their mechanical strength (first peak in the force/strain curve) (Qi et al., 2020).

#### 2.2.3 Dynamic gastrointestinal digestion in the NERDT

##### 2.2.3.1 Settings for gastrointestinal transit and digestive conditions of NERDT

**Fig. 1:**
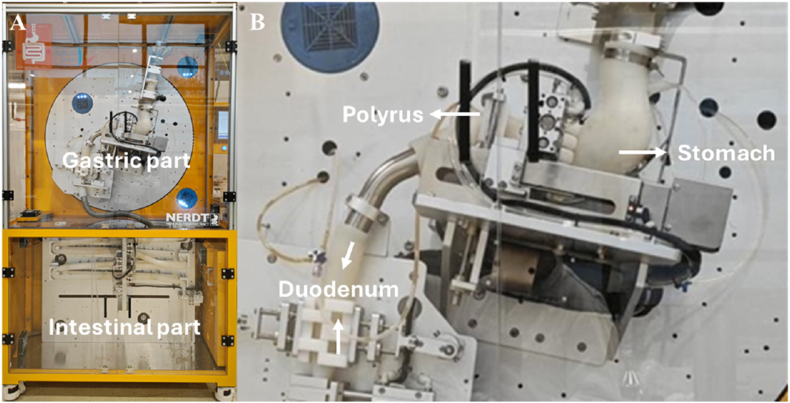
A picture of the whole system (A) and the gastric part (B) of NERDT.

The NERDT (Human adult model, Holland Green science Europe) was used to investigate the impact of size and density of the emulsion-alginate beads on the gastric emptying profile and intestinal lipolysis. The NERDT has an electro-mechanical driving rig to simulate the movement and mechanical force in stomach and peristalsis in the small intestine (Fig. 1). To better compare static and dynamic digestion, the digestive conditions (ratio of food to digestive fluids, enzyme activities, and bile salt concentration) in the stomach and small intestine of the NERDT were controlled to simulate the standardized INFOGEST conditions (Dupont et al., 2019).

To mimic the gastric emptying profile of our emulsion-alginate beads, we selected small emulsion-alginate beads (2 % alginate) as the reference. The relevant gastric emptying profile of this bead refers to the transit of alginate gel beads investigated by MRI reported by others (Hoad et al., 2009). The pylorus height was 5 mm, to mimic gastric sieving (Keet, 1962), and tilting direction of the stomach were adjusted to obtain theoretical gastric emptying conditions (Peng et al., 2021) that match the classic curve gastric emptying profiles of this beads (*t*_*0.5*_–50 min and *β* ∼ 1). The injection of simulated digestive fluids was chosen to match the *in vivo* passage profile: around 2 h for the stomach and a total of 3 h (Read et al., 1986; Yuen, 2010); all settings are in Table S1. Briefly, the total amount of volume ratio of gastric fluids emptied into small intestine and secreted intestinal fluids were controlled at 1:1 according to the settings of NERDT (see Fig. S1). Meanwhile, the dynamic pH, lipase activity and bile salt concentration in the intestine were controlled by adjusting SGF, SIF and NaOH to align with the INFOGEST conditions (Brodkorb et al., 2019).

##### 2.2.3.2 Dynamic digestion in the NERDT

Before use, emulsion-alginate beads were first washed with ultrapure water in a sieve (RETSCH Test Sieve, 200 mm) and then the surface water was removed by filter paper.

###### 2.2.3.2.1 Oral incubation

Next, 80 g of emulsion-alginate beads were mixed with 100 g of ultrapure water and 100 g of SSF to simulate oral digestion for 1 min as reported (Peng et al., 2021). This step was used to simulate the oral incubation without chewing (mimicking the size of food after chewing). The mixture was gently poured into the oesophagus part of the NERDT at the start of the digestion program.

###### 2.2.3.2.2 Gastrointestinal digestion

Simulated salivary fluid (SSF), simulated gastric fluid (SGF), and simulated intestinal fluid (SIF) were prepared based on the INFOGEST protocol (Brodkorb et al., 2019). Pepsin was added to the SGF at an initial activity of 2000 U/mL. The pH of SSF and SGF was adjusted to 7 and 2, respectively. The simulated gastric juice was added constantly to the gastric phase, and the dynamic pH profile was monitored (see 2.2.3.3). The SIF was added to digesta leaving the stomach in the duodenum phase (including emulsion-alginate beads). The initial lipase activity and bile salt concentration in SIF were 4500 U/mL and 22.5 mM, respectively (pH 7). In general, lipolysis starts after the emulsion-alginate beads pass the pylorus and enter the duodenum where they mixed with the SIF and neutralized by NaOH in NERDT.

##### 2.2.3.3 Analysis of gastric emptying

To analyse the emptied digesta, samples were taken after the pylorus every ∼5 min, and the gastric pH was measured. After that, the beads were gently filtered and weighed. The gastric retention ratio (*G*, %) was calculated from the mass of initially added beads (*I*_*beads*_, g) and the total of emptied beads (*E*_*beads*_, g):

Eq. 4 $$G=\frac{I_{beads}‐E_{beads}}{I_{beads}}\times 100$$

The Elashoff model (Eq. (5)) (Elashoff et al., 1982) was used to describe gastric retention over time using a power constant *β* and half emptying time (*t*_*0.5*_, min).

Eq. 5 $$G\left(t\right)=2^{-{\left(t\;/t_{0.5}\;\right)}^{\beta }\;}$$

To analyse bead structure and lipolysis, 15 mL digesta was collected at ∼0.5 and 2.5 m into the intestinal tube after 30, 60, 90, 120 and 180 min. Light microscopy (Zeiss Axioscope A1, Axiocam Mrc 5) at 10-time magnification was used to investigate the structure of the beads. The lipolysis degree of the digesta was measured by the HPLC as discussed below.

##### 2.2.3.4 Determination of lipolysis degree

The High Performance Liquid Chromatography with Charged Aerosol Detector (HPLC-CAD) (Vanquish, normal phase) was used in combination with a LC column (Column size: 250 × 3.0 mm I.D. with YMC-Pack PVA-Sil, Analytical Guard Cartridge (3.0 mm ID), 12 nm) (Moreau, 2009). The oil was extracted from the beads as previously described for oil content determination. Standards of FA, MAG, DAG and TAG (see materials) dissolved in heptane were added to reach 0.005–0.2 mg/mL. The extracted oil was diluted to the standard curve range and transferred into HPLC vials (1.50 mL Screw Neck Vial, BGB) with glass inserts (260 μL flat bottom glass insert, BGB). The two mobile phases were (A) heptane with 10 % isopropanol and 0.1 % glacial acetic acid and (B) heptane with 0.1 % glacial acetic acid. The column temperature was 25 °C, and samples (10 μl) were eluted at 0.43 ml/min using settings: 0–8 min, 90 % B; 8–28 min, linear decrease to 0 % B; 28–35 min, 0 % B; 35–37 min, linear increase to 90 % B; 37–40.2 min, 90 % B. After each analysis, the gradient pump was flushed with 90 % B for 15 min. The lipolysis degree (LD) was calculated based on the molar concentration (*C*) of free fatty acid (FFA), monoglyceride (MAG), diglyceride (DAG) and triglyceride (TAG) as measured by HPLC-CAD (Eq. (6)). Molecular weights (g/mol) were: 276, 350, 608, and 866, as reported previously (Corstens et al., 2018).

Eq. 6 $$\text{LD}\;\left(\%\right)=\frac{C_{FFA}}{3\times C_{TAG}+2\times {\;C}_{DAG}+C_{MAG}+C_{FFA}}\times 100$$

#### 2.2.4 Modelling the intestinal lipolysis of emulsion-alginate beads

**Fig. 2:**
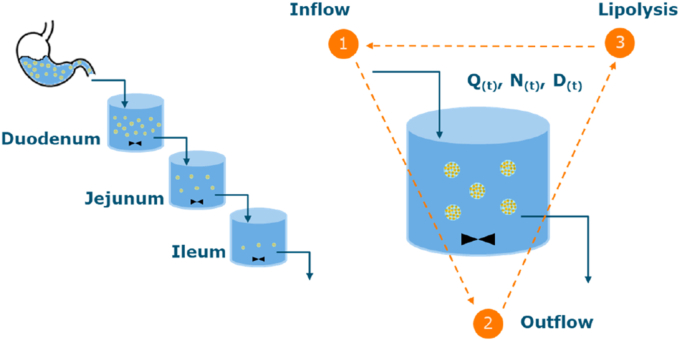
Schematic of model of dynamic intestinal lipolysis.

The gastric emptying profiles were combined with a lipolysis rate defined per oil droplet surface area (Wu et al., 2025) to predict the intestinal lipolysis profile. The intestinal phase consisted of 3 compartments representing duodenum, jejunum and ileum (Rivera del Rio et al., 2022). For each compartment, a balance equation was used to track 1) inflow and 2) outflow of substrate, and 3) extent of lipolysis (Fig. 2). We assume the digesta is perfectly mixed in the NERDT; but could not monitor this in our system.

The amount of oil emptied from the stomach (*Q*_*G(t)*_, μmol) was calculated based on the gastric emptying profiles (assuming no swelling or shrinking), total mass of beads (*M*_*beads*_, g), molecular weight of oil (*MW*_*oil*_ = 874 g/mol) and oil content (*C*_*oil*_, wt%) (Eqs. (7) and (8)).

Eq. 7 $$M_{oil\left(t\right)}=M_{beads\left(t\right)}\times C_{oil}$$

Eq. 8 $${\;Q}_{G}\left(t\right)=\left(2^{-{\left(t\;/t_{0.5}\;\right)}^{\beta }\;}\right)\times \frac{M_{oil\left(t\right)}}{{MW}_{oil}}\times 10^{6}$$

The initial size of the oil droplets in the emulsion-alginate beads was 18.2 μm (*D*_*0*_). The droplet size (*D,* μm) together with the number of droplets (*N*_*d*_) is used to calculate the available surface area (*A*_*d*_, m^2^) (Eq. (9)). The transit of digesta from stomach to duodenum and between the intestinal compartments were calculated by the residence time (duodenum: 30 min, jejunum: 60 min, ileum: 60 min) (Read et al., 1986; Yuen, 2010). After the inflow, the size (*D,* μm) of oil droplets were then averaged based on area (Sauter Mean Diameter) using both the existing droplets and the incoming droplets of initial size.

Eq. 9 $$A_{d}\left(t\right)=\pi \times {D\left(t\right)}^{2}\times N_{d}\left(t\right)\times 10^{-12}$$

##### 2.2.4.1 Lipolysis calculation of intestinal compartments

During transit in duodenum, jejunum and ileum, lipolysis will take place, which reduces the volume of the droplets, and thus the surface area available for lipolysis. The lipolysis rate (μmol·s^−1^) follows from the surface area and the lipolysis rate constant (*k*, 8.5 μmol m^−2^·s^−1^) (Wu et al., 2025), which is used to calculate the quantity of oil in the compartments at different times.

Eq. 10 $${\;Q}_{lipo}\left(t\right)=A\left(t\right)\times k$$

Eq. 11 $$\frac{dQ\left(t\right)}{dt}={\;Q}_{in}\left(t\right)-{\;Q}_{out}\left(t\right)-{\;Q}_{lipo}\left(t\right)$$

##### 2.2.4.2 Lipolysis degree and FFA release

In Eq. (6), we calculate the lipolysis degree of digesta collected from the NERDT by the composition. The cumulative lipolysis degree (%, up to 67 %) in each intestinal compartment (*in silico* model) is calculated by the reduction in the molar quantity of triglycerides. Besides, the total FFA release rate (μmol·s^−1^) in each compartment is calculated (Eq. (10)). The droplet size reduction was calculated based on lipolysis rate. To compare with the FFA release (%) with others, the cumulative FFA release in each compartment was calculated from the ratio of released to total titratable FFAs (two FFA for one triglyceride (TAG), up to 100 %).

#### 2.2.5 Statistics

All experiments were conducted in triplicate. Microsoft® Excel® Office 365 (Redmond, Washington, USA) was used for primary data analysis and lipolysis modelling. Matlab R2023b was used for fitting the gastric emptying profile. For significance, SPSS 28 (IBM, Armonk, New York, USA) was used with one-way ANOVA with Turkey's post-hoc test. The results are given as means ± standard deviation.

## 3 Results and discussion

Characteristics of the emulsion-alginate beads are described in section 3.1, and their gastric emptying in section 3.2. This is used as input for the model and compared to experimental results in section 3.3.

### 3.1 Characterization of the emulsion-alginate beads

We characterized the particle size, calculated density and measured mechanical strength of emulsion-alginate beads (Table 1). The sizes of small, medium and large emulsion-alginate beads are around 0.9, 2.4 and 3.2 mm, respectively. The oil content of emulsion-alginate beads (12–32 %) decreased with increasing alginate concentration, which is in line with the density increase calculated with Eq. (3) and previous reports (Lin et al., 2020; Wu et al., 2024), and visual observations of beads in simulated gastric juice (density 1.024 g/mL; see Fig. S2). In addition, based on the fracture force measurements, the strength of emulsion-alginate beads increased with increasing initial alginate concentration and with bead size. Higher strength may delay the gastric emptying of gel beads due to the interception of less deformable larger beads (Wickham et al., 2012). Our emulsion-alginate beads that are relatively small were structurally stable (remained intact).

**Table 1:** Characterization of the initial emulsion-alginate beads.

Name	Initial alginate concentration	Particle size	Oil content	Calculated density	Fracture force
	%	mm	%	g/mL	N
Small 1 %	1	0.97 ± 0.07	31.6 ± 0.5d	1.003 ± 0.000a	0.22 ± 0.03a
Small 2 %	2	0.81 ± 0.08	21.2 ± 1.2bc	1.018 ± 0.001a	0.45 ± 0.06a
Small 3 %	3	0.97 ± 0.06	12.5 ± 0.9a	1.027 ± 0.000b	0.83 ± 0.09b
Medium 1 %	1	2.41 ± 0.10	27.9 ± 7.0cd	1.006 ± 0.006a	0.98 ± 0.11bc
Medium 2 %	2	2.56 ± 0.10	18.1 ± 1.5 ab	1.020 ± 0.001b	1.21 ± 0.09c
Medium 3 %	3	2.37 ± 0.11	14.6 ± 1.4 ab	1.027 ± 0.000c	2.18 ± 0.19d
Large 2 %	2	3.21 ± 0.23	15.5 ± 0.9 ab	1.021 ± 0.000bc	3.71 ± 0.44f

Different letters present a significant difference within the column following one-way ANOVA with Turkey's post-hoc test.

### 3.2 Gastric emptying of emulsion-alginate beads

**Fig. 3:**
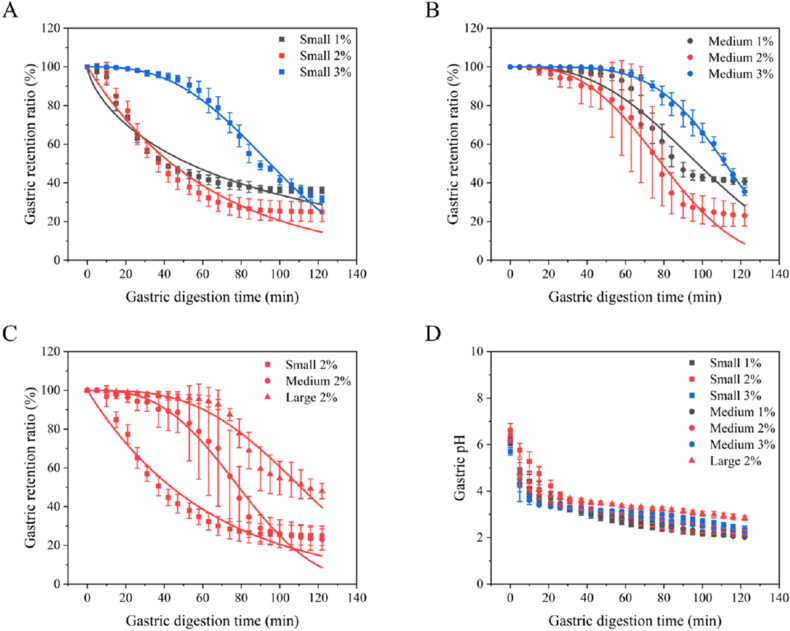
Gastric retention of emulsion-alginate beads with different alginate concentration (1 % black, 2 % red, 3 % blue) and A) small and B) medium particle size, C) and different sizes at 2 % alginate. D) gastric pH.

The gastric emptying of the emulsion-alginate beads was measured in the NERDT. The emptying profiles (Fig. 3A, B and C) were fitted with the Elashoff model (Elashoff et al., 1982) to obtain values for *β*, and *t*_*0.5*,_ min (Table 2). Large particles showed delayed gastric emptying (Fig. 3C); the half-emptying time increased from 44 min (for 0.8 mm beads) to 111 min (for 3.2 mm beads) and delayed lag time *β* from 1.0 to 3.1 at similar bead density (∼1.02 g/mL for both 2 % alginate beads) (Table 2). The gastric pH profile was similar for all samples, decreasing from around pH 6 to ∼2.5 after 2 h gastric incubation (Fig. 3D). The decreasing pH in the stomach induced minimal shrinking of the emulsion-alginate beads.

**Table 2:** Behaviour of the beads in simulated gastric fluid and fitting parameters of the gastric retention curves.

	*Mixing behaviour*	*β*	*t*_*0.5*_
			min
Small 1 %	Floating	0.7 ± 0.0a	52.8 ± 3.5a
Small 2 %	Floating (slower)	1.0 ± 0.1a	43.8 ± 5.9a
Small 3 %	Sinking	2.8 ± 0.3b	94.9 ± 3.4bc
Medium 1 %	Floating	2.7 ± 0.3b	97.5 ± 3.7bc
Medium 2 %	Floating (slower)	2.9 ± 0.9b	78.8 ± 15.2b
Medium 3 %	Sinking	4.6 ± 0.8c	111.9 ± 2.2c
Large 2 %	Floating	3.1 ± 0.3b	111.0 ± 7.9c

Different letters present a significant difference within the column following one-way ANOVA with Turkey's post-hoc test.

Small (<1 mm) floating particles are emptied like a liquid (*β* ≤ 1, *t*_*0.5*_∼50 min) with no lag time (Fig. 3 A) (Goyal et al., 2019), while higher density particles show significantly delayed gastric emptying and increased lag time (*β* = 2.8, *t*_*0.5*_ = 94 min) (Fig. 3B). Sinking particles are less affected by gastric turbulence and have less chance of reaching the pylorus (Goyal et al., 2019). Particles having a similar density to the gastric fluid can be more easily suspended by turbulence (Gu et al., 2020), after which the pylorus plays a critical role in gastric sieving. In work of others, similar effects have been found in an *in vivo* study in which labelled spheres of 1 mm showed faster emptying (no lag time) than those of 2.4 and 3.2 mm (density 1 g/mL) (Meyer et al., 1988). For food particles, it was found that a smaller size (<2–3 mm) corresponds to fast gastric emptying (Goyal et al., 2019). Although further verification is needed, the half gastric emptying time of emulsion-alginate beads in the NERDT is close to the *in vivo* results (Hoad et al., 2009). This suggests that the stomach model of NERDT is suitable for investigating the gastric transit of food.

It also highlights the importance of the density of food particles relative to the density of the gastric fluid for their likelihood to remain suspended. This, together with food size relative to pylorus size (gastric sieving) determines the gastric emptying profile.

### 3.3 Dynamic intestinal digestion of emulsion-alginate beads

#### 3.3.1 *In silico* modelling of dynamic intestinal digestion of emulsion-alginate beads

**Fig. 4:**
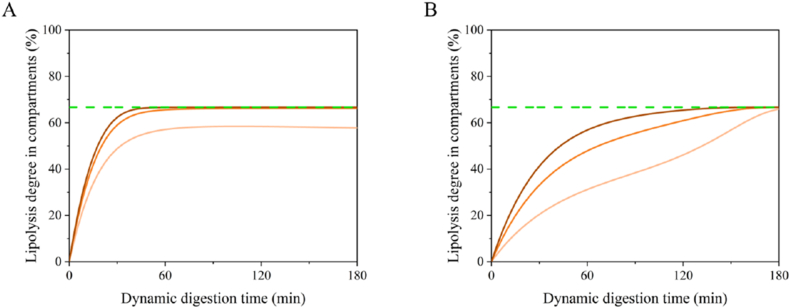
Modelling of the lipolysis degree (%) of small 1 % (A) and medium 3 % (B) emulsion-alginate bead in duodenum (pale orange), jejunum (orange) and ileum (brown) calculated as released FFAs compared to the present FA in the compartment at that timepoint. Green dashed lines represent the maximum lipolysis degree (2 FFA per 3 FFA on the TAG,67 %).

The impact of the gastric emptying profiles (Fig. 3) on intestinal lipolysis of emulsion-alginate beads was modelled based on earlier work (Wu et al., 2025). it is clear that emulsion-alginate beads with fast gastric emptying (Small 1 %) showed fast lipolysis and reached a plateau after 60 min (Fig. 4A). For beads with slow emptying, the lipolysis degree increased much slower (Medium 3 %), also because of the lag phase (Fig. 4B). These distinct lipolysis profiles indicated that slower gastric emptying leads to delayed lipolysis (spread out over a longer time), which relates to the size and density of the beads as we discussed in 3.2. The modelling results show that the gastric emptying profile dictates intestinal lipolysis by controlling the addition of oil to the intestinal environment. In general, 63–79 % of cumulative FFA release occurs in the duodenum, approximately 17 % in the jejunum, and about 3 % in the ileum (2 fatty acids can released from 1 triglyceride), which is in line with *in vivo* studies that show that 50–70 % of FFA are released in the duodenum (Bauer et al., 2005).

#### 3.3.2 Dynamic intestinal digestion in the NERDT

**Fig. 5:**
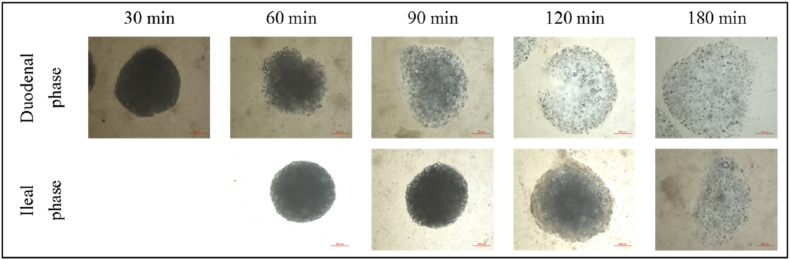
Microscope pictures of emulsion-alginate beads collected from different intestinal parts, the scale bars are 200 μm.

Before comparing the model with measurements, we discuss bead integrity. The beads gradually swell during intestinal digestion, both in the duodenum and ileum (Fig. 5) due to changes in pH (Fig. S3). Na^+^ and Ca^2+^ ions compete for binding sites on the alginate (Martinsen et al., 1989) which leads to the observed swelling behaviour. During 2–3 h dynamic intestinal digestion, the emulsion-alginate beads do not break down, unlike in *in vitro* digestion (Wu et al., 2024). This is likely due to the difference between constant shear force (Vardakou et al., 2011) in static digestion and the intestinal peristalsis in the NERDT. The gastric pH decreased within half an hour to pH 3 like in static protocols, so ion exchange in the stomach is not expected to explain differences.

**Fig. 6:**
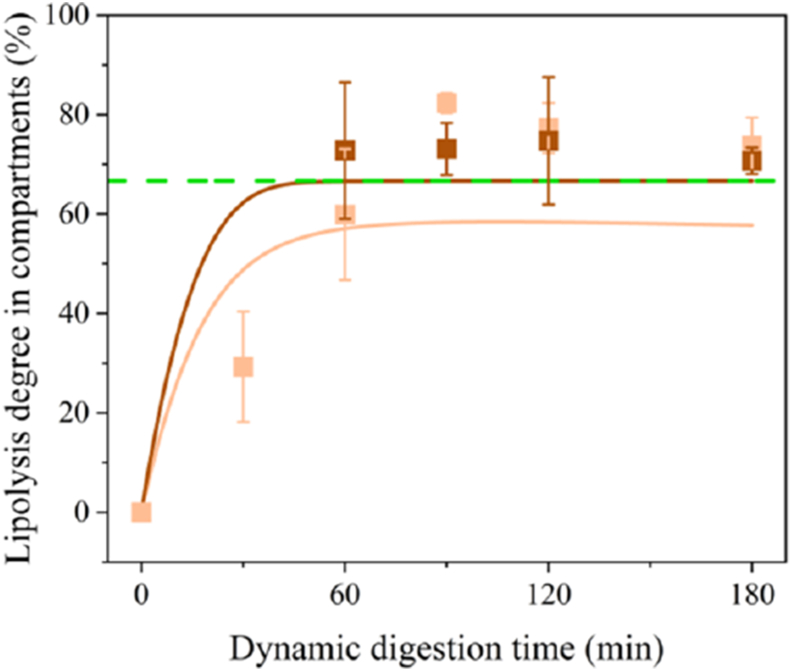
Lipolysis degree (%) of small 1 % emulsion-alginate beads in compartments during dynamic intestinal digestion. Experimental results (squares) obtained by HPLC and model predictions (lines) are shown for the duodenal phase (pale orange) and the ileal phase (brown) calculated as released FFAs compared to the present FA in the compartment at the time point. Error bars represent the standard deviation of measurements taken in triplicate. The dashed green line indicates theoretical complete lipolysis (2 released FFAs per 3 FA on TAG, 67 %).

The degree of intestinal lipolysis of Small 1 % emulsion-alginate bead was measured in the NERDT duodenal (0.5 m) and ileal (2.5 m) phase (see Fig. 6). The degree of lipolysis in the ileum increased to around the theoretical maximum value after 60 min of digestion, while in the duodenum it increased to approximately 58 %, which matches well with the model. Experimental differences in lipolysis degree (Fig. 6) are most probably due to heterogenous mixing in the duodenum section (Schulze, 2015). The least squares fit of the experimental data to the modelled curves yielded an R^2^ of 0.790 for the duodenal phase and 0.998 for the ileal phase (Fig. 6). This indicates that the lipolysis model's predictive accuracy is lower for the duodenum, suggesting that the model's uncertainty in this specific part of the NERDT system needs to be addressed. Future work should focus on validating the mathematical model as well as the *in vitro* NERDT model to further improve the duodenal phase and enhance its ability to predict *in vivo* lipid digestion more accurately.

**Fig. 7:**
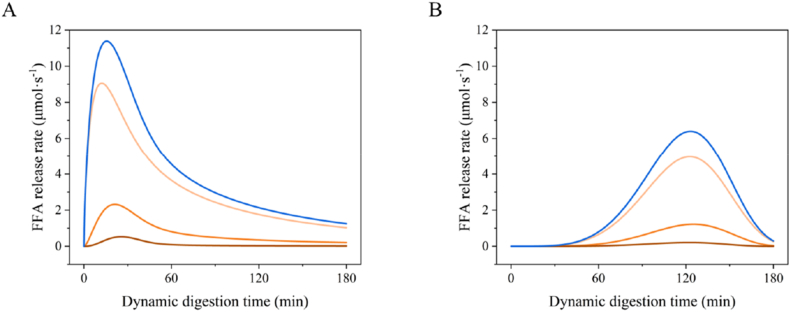
*The modelled FFA release* rate (*μmol·s*^*−1*^) of Small 1 % *(A) and Medium 3 % (B) emulsion-alginate bead in duodenum (pale orange), jejunum (orange), ileum (brown) and total intestine (blue).*

The rate of FFA release (μmol·s^−1^) was calculated based on the available surface area in time per compartment using the equations in section 2.2.4. Fig. 7 shows the FFA release rate per compartment, thus providing insight into the likely location of absorption. Small 1 % beads had a much faster FFA release rate within the first hour, due to the faster gastric emptying profile, whereas medium 3 % beads showed a delayed peak at 2 h due to slower gastric emptying. The simulation of the FFA ready to be absorbed of other beads are shown in Fig. S4. These findings suggest that the gastric emptying profiles of emulsion–alginate beads can be used to modulate FFA release, and through that also their absorption.

From the work presented here, it is clear that transit and mixing of food greatly affect lipolysis along the intestinal length. This gastric emptying–determined intestinal lipolysis provides a valuable insight for designing controlled release vehicles, not just for fatty acids but also lipophilic bioactive compounds.

## 4 Conclusion

Using the dynamic digestion system NERDT, we demonstrated how particle size and density influence the gastric emptying behaviour of emulsion-alginate beads and subsequently impact intestinal lipolysis. The particle size and density determine half gastric emptying time (44–112 min) and shape of emptying curve (*β*: 0.7 to 4.6). High particle density and size resulted in slower emptying and, hence, slower lipolysis. The observed experimental effects were adequately described using a model combining gastric emptying profiles with lipolysis kinetics based on available oil surface area. These findings indicate that by tuning density and size of emulsion-alginate beads, distinct release profiles can be created that potentially enable targeted delivery in specific sites of the GI tract. These findings are expected to be instrumental in designing functionalised food structures, as well as in bringing *in vitro* and *in vivo* studies closer together.

## Author statement

Lingfeng Wu: Conceptualization, Investigation, Methodology, Analysis, Writing - Original Draft, Karin Schroen: Conceptualization, Supervision, Writing - Review & Editing, Meinou Corstens: Conceptualization, Methodology, Supervision, Writing - Review & Editing.

## Declaration of competing interest

The authors declare that they have no known competing financial interests or personal relationships that could have appeared to influence the work reported in this paper.
