# Supplementary material for: Size- and density-dependent gastric emptying of emulsion-alginate beads for tailored in vitro intestinal lipolysis
Source: Curr Res Food Sci. 2025 Dec 15;12:101279. doi: 10.1016/j.crfs.2025.101279 (PMC12794519; doi:10.1016/j.crfs.2025.101279)
Supplement: Multimedia component 1 [file mmc1.docx]

Supplementary information for

**Size- and density-dependent gastric emptying of emulsion-alginate beads for tailored *in vitro* intestinal lipolysis**

Lingfeng Wu^1^, Karin Schroën^1^, Meinou Corstens*^1^

*Corresponding author: meinou.corstens@wur.nl

^1^Wageningen University, Department of Agrotechnology & Food Sciences, Laboratory of Food Process Engineering, Bornse Weilanden 9, 6708 WG Wageningen, The Netherlands.

*Table S1 Program of gastric and intestinal digestion in NERDT*

| Oesophagus valve Operating Parameter (Time: min) | | | | | | |
| --- | --- | --- | --- | --- | --- | --- |
| Stage | 1 | 2 | 3 | 4 | 5 | 6 |
| Open time | 4.9 |  |  |  |  |  |
| Close time | 0.1 |  |  |  |  |  |
| Running time | 180 |  |  |  |  |  |
| Oesophagus Device Operating Parameter (Speed: rpm, Time: min) | | | | | | |
| Stage | 1 | 2 | 3 | 4 | 5 | 6 |
| Open upper oesophagus clamp | 0.1 |  |  |  |  |  |
| Open lower oesophagus clamp | 0.1 |  |  |  |  |  |
| Shaking rate of oesophagus | 50 |  |  |  |  |  |
| Running time | 5 |  |  |  |  |  |
| Gastric fluid feeding device Operating Parameter (Speed: mL/min, Time: min) | | | | | | |
| Stage | 1 | 2 | 3 | 4 | 5 | 6 |
| Speed | 2.2 | 1 |  |  |  |  |
| Time | 124 | 26 |  |  |  |  |
| Stomach Tilting Device Operating Parameter (Speed: °/min, Time: min) | | | | | | |
| Stage | 1 | 2 | 3 | 4 | 5 | 6 |
| Speed | 6 | -0.5 | -0.2 | -0.5 | -1 | 0 |
| Time | 5 | 5 | 10 | 30 | 40 | 60 |
| Gastric peristalsis Device Operating Parameter (Speed: mm/min, Time: min) | | | | | | |
| Stage | 1 | 2 | 3 | 4 | 5 | 6 |
| Forward Speed | 500 |  |  |  |  |  |
| Reset Speed | 600 |  |  |  |  |  |
| Running time | 150 |  |  |  |  |  |
| Pylorus Device Operating Parameter (Speed: mm/min, Time: min) | | | | | | |
| Stage | 1 | 2 | 3 | 4 | 5 | 6 |
| Width of release | 5 |  |  |  |  |  |
| Speed | 100 |  |  |  |  |  |
| Number of stomach squeezes | 15 |  |  |  |  |  |
| Running time | 150 |  |  |  |  |  |
| Intestinal fluid feeding device Operating Parameter (Speed: mL/min, Time: min) | | | | | | |
| Stage | 1 | 2 | 3 | 4 | 5 | 6 |
| Speed | 0.5 | 4 | 0.1 |  |  |  |
| Time | 30 | 70 | 50 |  |  |  |
| Intestinal peristalsis Device Operating Parameter (Speed: mm/min, Time: min) | | | | | | |
| Stage | 1 | 2 | 3 | 4 | 5 | 6 |
| Speed | 100 |  |  |  |  |  |
| Time | 180 |  |  |  |  |  |
| Duodenum valve Operating Parameter (Time: min) | | | | | | |
| Stage  Open time  Close time  Running time | 1 | 2 | 3 | 4 | 5 | 6 |
| Open time | 5 | 5 |  |  |  |  |
| Close time | 0 | 0.5 |  |  |  |  |
| Running time | 5 | 175 |  |  |  |  |
| Intestine valve Operating Parameter (Time: min) | | | | | | |
| Stage  Open time  Close time  Running time | 1 | 2 | 3 | 4 | 5 | 6 |
| Open time | 30 |  |  |  |  |  |
| Close time | 0.1 |  |  |  |  |  |
| Running time | 180 |  |  |  |  |  |
| Intestine pH adjustment (Speed: mL/min, Time: min) | | | | | | |
| Stage | 1 | 2 | 3 | 4 | 5 | 6 |
| Speed | 0 | 0.5 | 0.8 | 1 | 0.8 |  |
| Running time | 15 | 15 | 59 | 21 | 19 |  |

*
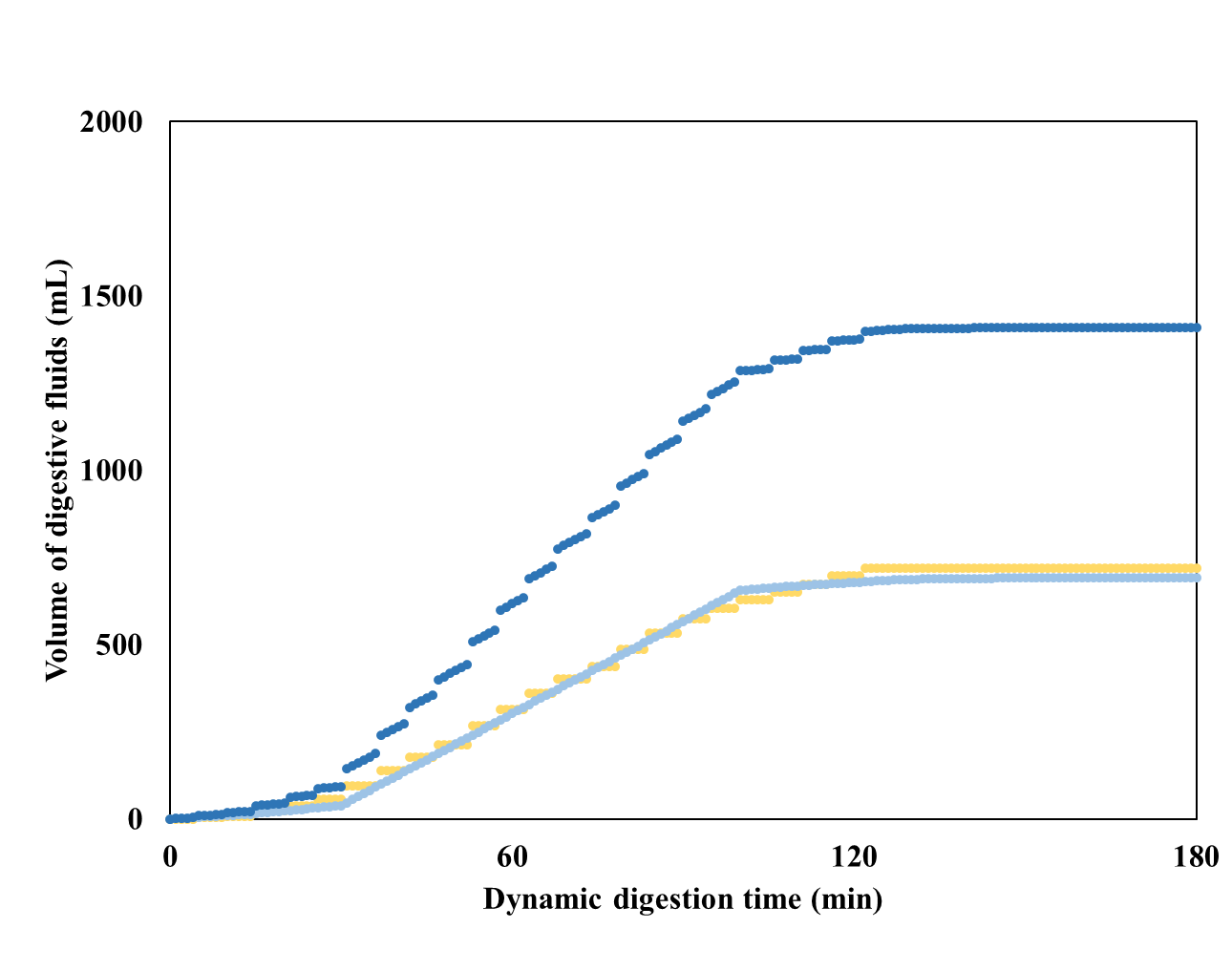
*

*Figure S1 Volume (mL) of digestive fluids in the NERDT: gastric secreted (yellow), intestinal secreted (light blue) and total added intestinal fluid (blue).*


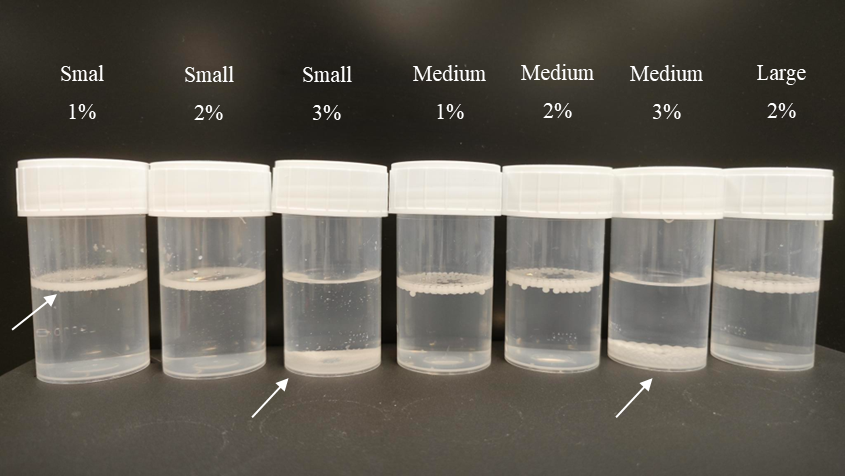


*Figure S2 Behaviours of emulsion-alginate beads in SGF (pH=2) incubation.*


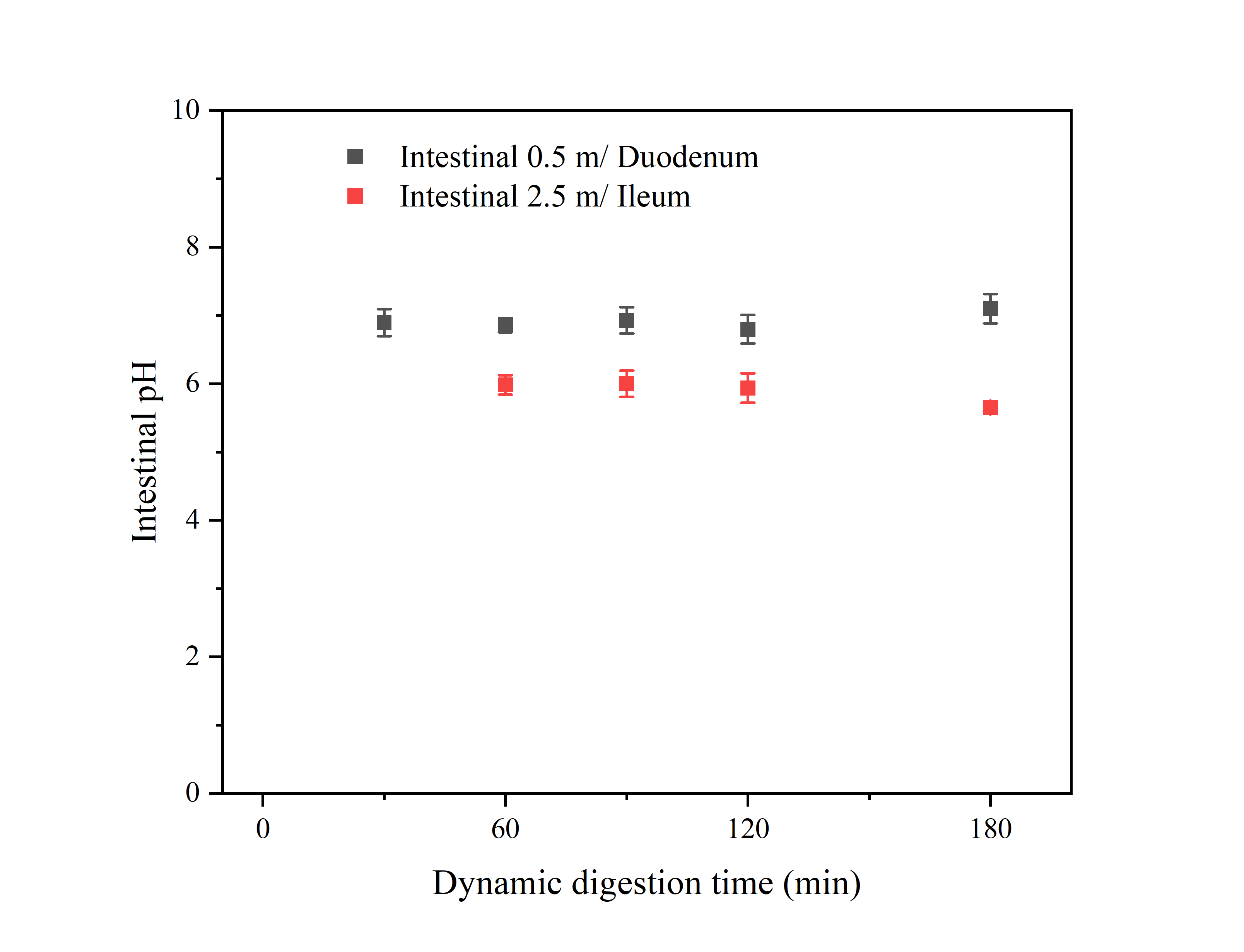


*Figure S3 Intestinal pH at different intestinal locations (black squares: 0.5 m; read squares: 2.5 m).*

*
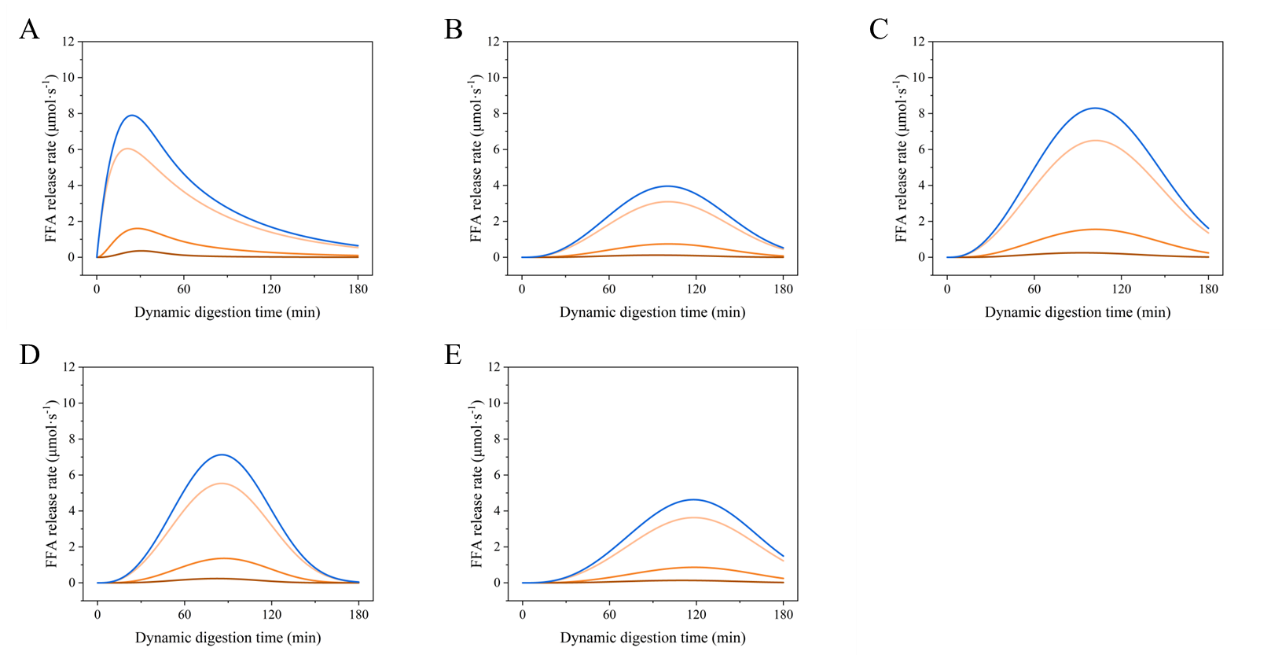
*

*Figure S4 The modelled FFA release rate (μmol·s^-1^) of Small 2% (A), Small 3% (B), Medium 1% (C), Medium 2% (D), Large 2% (E) emulsion-alginate bead in duodenum (pale orange), jejunum (orange), ileum (brown) and total intestine (blue).*
